# Supplementary material for: Resolving the full spectrum of human genome variation using Linked-Reads
Source: Genome Res. 2019 Apr;29(4):635–45. doi: 10.1101/gr.234443.118 (PMC6442396; doi:10.1101/gr.234443.118)
Supplement: Supplemental Material [file supp_gr.234443.118_Supplemental_longranger-2.2.zip › longranger-2.2.tar.bz2!/lr2.2-public/lib/stan/cmdstan/stan/doc/api/html/assign_8hpp_source.html]

Stan: src/stan/math/matrix/assign.hpp Source File


|  |  |
| --- | --- |
|  | Stan  2.5.0  probability, sampling & optimization |


- Main Page
- Related Pages
- Namespaces
- Classes
- Files

- File List
- File Members

All Classes Namespaces Files Functions Variables Typedefs Enumerations Enumerator Friends Macros Pages

- src
- stan
- math
- matrix

assign.hpp

Go to the documentation of this file.

1 #ifndef STAN\_\_MATH\_\_MATRIX\_\_ASSIGN\_HPP

2 #define STAN\_\_MATH\_\_MATRIX\_\_ASSIGN\_HPP

3

4 #include <vector>

5 #include <sstream>

6 #include <stdexcept>

7

8 #include <stan/math/matrix/Eigen.hpp>

9 #include <stan/math/error\_handling/matrix/check\_matching\_sizes.hpp>

10 #include <stan/math/error\_handling/matrix/check\_matching\_dims.hpp>

11

12

13 #include <iostream>

14

15 namespace stan {

16

17  namespace math {

18

19  // Recursive assignment with size match checking and promotion

20

35  template <typename LHS, typename RHS>

36  inline void

37  assign(LHS& lhs, const RHS& rhs) {

38  lhs = rhs;

39  }

40

61  template <typename LHS, typename RHS, int R1, int C1, int R2, int C2>

62  inline void

63  assign(Eigen::Matrix<LHS,R1,C1>& x,

64  const Eigen::Matrix<RHS,R2,C2>& y) {

65  std::stringstream ss;

66  ss << "error in call to assign(Eigen::Matrix,Eigen::Matrix)"

67  << "; shapes must match, but found"

68  << " R1=" << R1

69  << "; C1=" << C1

70  << "; R2=" << R2

71  << "; C2=" << C2;

72  throw std::domain\_error(ss.str());

73  }

74

92  template <typename LHS, typename RHS, int R, int C>

93  inline void

94  assign(Eigen::Matrix<LHS,R,C>& x,

95  const Eigen::Matrix<RHS,R,C>& y) {

96  stan::math::check\_matching\_dims("assign(%1%)",x,"x",

97  y,"y",(double\*)0);

98  for (int i = 0; i < x.size(); ++i)

99  assign(x(i),y(i));

100  }

101

120  template <typename LHS, typename RHS, int R, int C>

121  inline void

122  assign(Eigen::Block<LHS> x,

123  const Eigen::Matrix<RHS,R,C>& y) {

124  stan::math::check\_matching\_sizes("assign(%1%)",x,"x",

125  y,"y",(double\*)0);

126  for (int n = 0; n < y.cols(); ++n)

127  for (int m = 0; m < y.rows(); ++m)

128  assign(x(m,n),y(m,n));

129  }

130

131

151  template <typename LHS, typename RHS>

152  inline void

153  assign(std::vector<LHS>& x, const std::vector<RHS>& y) {

154  stan::math::check\_matching\_sizes("assign(%1%)",x,"x",

155  y,"y",(double\*)0);

156  for (size\_t i = 0; i < x.size(); ++i)

157  assign(x[i],y[i]);

158  }

159

160

161  }

162 }

163 #endif

check\_matching\_dims.hpp

check\_matching\_sizes.hpp

stan::math::check\_matching\_dims

bool check\_matching\_dims(const char \*function, const Eigen::Matrix< T1, R1, C1 > &y1, const char \*name1, const Eigen::Matrix< T2, R2, C2 > &y2, const char \*name2, T\_result \*result)

**Definition:** check\_matching\_dims.hpp:15

Eigen.hpp

stan::math::assign

void assign(LHS &lhs, const RHS &rhs)

Copy the right-hand side's value to the left-hand side variable.

**Definition:** assign.hpp:37

stan::math::check\_matching\_sizes

bool check\_matching\_sizes(const char \*function, const T\_y1 &y1, const char \*name1, const T\_y2 &y2, const char \*name2, T\_result \*result)

**Definition:** check\_matching\_sizes.hpp:15


---

[ Stan Home Page ]

*© 2011–2014,
Stan Development Team.*
